# Does response shift impact interpretation of change even among scales developed using item response theory?

**DOI:** 10.1186/s41687-019-0162-x

**Published:** 2020-01-23

**Authors:** Carolyn E. Schwartz, Brian D. Stucky, Wesley Michael, Bruce D. Rapkin

**Affiliations:** 1 DeltaQuest Foundation, Inc., 31 Mitchell Road, Concord, MA 01742 USA; 2 Departments of Medicine and Orthopaedic Surgery, Tufts University Medical School, Boston, MA USA; 3 Los Alamos National Laboratory, Los Alamos, NM USA; 4 Rare Patient Voice, LLC, Towson, MD USA; 5 Department of Epidemiology and Population Health, Division of Community Collaboration & Implementation Science, Albert Einstein College of Medicine, Bronx, NY USA

**Keywords:** Item response theory, Classical test theory, Residual modeling, Response shift, Appraisal, Life events

## Abstract

**Background:**

Response-shift effects impact the interpretation of change in quality-of-life (QOL) measures developed with classical test theory (CTT) methods. This study evaluated the impact of response shift on measures developed using Item Response Theory (IRT), as compared to CTT.

**Methods:**

Chronically ill patients and caregivers (*n* = 1481) participated in a web-based survey at baseline and 17 months later. Patients completed the IRT-based PROMIS-10; NeuroQOL Applied Cognition, Positive Affect & Well-Being short-forms; and the CTT-based Ryff Environmental Mastery subscale. Response-shift effects were evaluated using regression residual modeling and the QOL Appraisal Profile-v2. The sample was divided into positive and negative catalyst groups on the basis of marital, work, job-status, and comorbidity change. Regression models predicted residualized QOL change scores as a function of catalysts and appraisal changes.

**Results:**

In this sample 859 (58%) reported a catalyst. No catalyst was associated with change in scales developed using IRT, but positive work change was associated with the CTT-based measure. Catalyst variables were associated with changes in appraisal, which in turn were related to all outcomes, particularly for global mental health after a positive work-change.

**Conclusions:**

Appraisal processes are relevant to interpreting IRT measures, particularly for global mental health in the face of life changes.

## Introduction

**Fig. 1:**
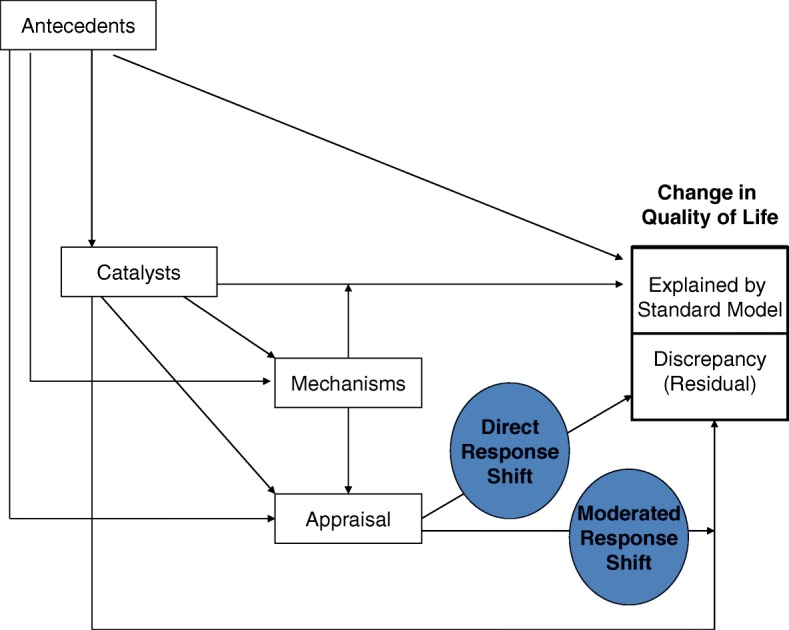
The Rapkin and Schwartz theoretical model. This model tests for response-shift effects by predicting residualized QOL change. Antecedents are adjusted as covariates in the “standard QOL model”, and the unexplained variance (residuals) is modeled as a function of catalysts and appraisal processes. Main effects of appraisal reflect direct response shift, and catalyst-by-appraisal interaction effects reflect moderated response shift

Research on a broad range of patient populations has suggested that response-shift effects can attenuate estimates of treatment benefit [1–8] and can explain paradoxical findings of improved quality of life (QOL) despite objectively poor functional status [9–16]. Response-shift theory [17, 18] predicts that when people experience a health-state change or *catalyst*, they may change their internal standards, values, or conceptualization of QOL (Fig. 1). These response-shift effects are influenced by stable characteristics of the individual (antecedents); as well as cognitive, behavioral, or affective processes (mechanisms). Appraisal processes are the basis for response shift: Response shift is inferred when appraisal changes explain the discrepancy between expected and observed QOL, given the catalysts experienced [18]. Appraisal can directly influence QOL change (direct response shift) and can moderate the impact of the catalyst (moderated response shift) on QOL change.

Response shift research using the direct assessment of appraisal [19, 20] focuses on individual-level change in how respondents think about QOL and has revealed important differences in health outcomes and resilience over time [21–26]. This approach enables a descriptive understanding of adaptation processes, and can point to useful directions for clinical intervention. Recent developments of practical measures of appraisal processes allow for individual-level analysis that characterizes the underlying cognitive processes connoted by response-shift effects [19, 20].

Many researchers familiar with methods of response shift detection based on lack of measurement invariance over time [27, 28] might reasonably assume that measures developed using item-response theory (IRT) [29] would be less subject to response shift. The logic is that IRT single-domain scales are designed to be unidimensional and, based on the probabilistic nature of the models, would have similar item characteristics across samples. Although IRT single-domain scales have generally been developed using cross-sectional data only, it would be reasonable to assume that the psychometric characteristics of IRT-based scales should be stable over time. They would thus be less subject to response shift effects as detected by measurement-invariance methods (e.g., Structural Equation Modeling). The motivation for this paper relates to differences in the strictness and rigor by which items are selected using IRT versus CTT. The IRT criteria for selecting items emphasize unidimensionality and are more rigorous and stricter than is the case with CTT. Those criteria ought to have the effect of being conceptually more linked to one another so may be reducing construct representation for the sake of internal consistency. The methods for evaluating differential item functioning (DIF) over time further enable selecting items whose characteristics are similar across sample or time, thereby reducing the likelihood of factorial variance and with it the likelihood of detecting certain types of response shift. Indeed, to the extent that items demonstrate DIF, they would also be more subject to appraisal differences and therefore to response-shift effects. Further, DIF methods may not be sufficient to detect response shift, not only because they focus on differences in item response in cross-sectional data due to stable characteristics of the individual (e.g., demographics), but also because response shift is by definition a longitudinal phenomenon (i.e., adaptation effects over time). Nonetheless, even if items do not demonstrate DIF, they may still be subject to appraisal differences and response-shift effects because these are part of adapting to change. Thus, even with the most measurement-invariant measures, these hallmarks of the human condition would be expected.

Thus, despite lack of evidence for response shift using measurement-invariance methods, individuals’ understanding or experience of the latent trait can still change. Appraisal necessarily always occurs whenever individuals rate their QOL (i.e., they are thinking about something relevant to the questions they are answering). Individuals need not appraise QOL in the same way at different times of measurement even when item or scale characteristics remain stable (i.e., same overall factor structure, same factor loadings, same inter-item correlations, etc.). In fact, as many studies observing homeostasis of QOL scores across the course of illness suggest, people actually maintain a QOL set point across changing health status or exposure to catalysts by changing their appraisal [21–23, 30, 31].

**Fig. 2:**
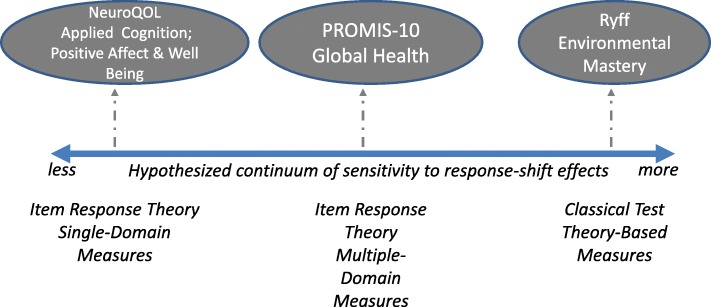
Hypothesized Continuum of Sensitivity to Response-Shift Effects. We posit that there is a continuum of measurement sensitivity to response-shift effects, with the least sensitive being measures developed using IRT methods with the intention of developing a specific and unidimensional measure of a construct (IRT single-domain measures). Measures hypothesized to be most sensitive to response-shift effects would be developed with a focus on alpha reliability and construct validity that follow logical arguments but have relatively fewer quantitative metrics of model fit and item function than IRT (CTT-based measures). In between these two extremes are measures that use IRT methods for calibration and item selection but which seek to be general tools for measuring QOL rather than measures of unidimensional constructs of domains (IRT multiple-domain measures).

This study investigates whether there are differences in sensitivity to response-shift effects among measures developed in different ways. We propose the distinction shown in Fig. 2: that there is a continuum of measurement sensitivity to response-shift effects, with the least sensitive generally being measures developed using IRT methods with the intention of developing a specific and unidimensional measure of a construct that would maximize internal consistency reliability (*IRT single-domain measures*). Measures hypothesized to be most sensitive to response-shift effects (*CTT-based measures*) would, on the one hand, be developed with a focus on alpha reliability and construct validity that follow logical arguments. Such measures would, however, have relatively fewer quantitative metrics. In fact, CCT-based measures do not have the benefit of model-fit statistics and item-function curves that IRT does. In between these two extremes on the continuum are measures that use IRT methods for calibration and item selection but which seek to be general tools for measuring QOL (i.e., maximizing band width) rather than measures of unidimensional constructs of domains (*IRT multiple-domain measures*). The analysis presented here will examine the extent to which IRT-unidimenstional measures demonstrate response shift in terms of responsiveness to catalysts, as well as their association with changes in appraisal. We hypothesize that compared to CTT-based and IRT multiple-domain measures, IRT single-domain measures will be less responsive to catalysts but similar in how much appraisal explains variance in change scores over time.

## Methods

### Sample

This secondary analysis utilized data collected from Rare Patient Voice, LLC and WhatNext panels, with a heterogeneous grouping of chronic health conditions (see www.rarepatientvoice.com). Eligible participants were patients with a chronic medical condition or their caregivers of age 18 years or older, and able to complete an online questionnaire.

### Procedure and design

A web-based survey was administered twice (baseline, follow-up) using the Health Insurance Portability and Accountability Act (HIPAA)-compliant, secure SurveyGizmo engine (www.surveygizmo.com). (See [32] for full description of methods.) The study was reviewed and approved by the New England Review Board (NEIRB#15–254), and all participants provided informed consent.

### Measures

**QOL** was assessed using the following patient-reported outcomes (PROs): The IRT single-domain PROs included the *NeuroQOL Applied Cognition General Concerns* and *Executive Function short-forms* [33]; and the *NeuroQOL Positive Affect & Well-Being* [33]. The IRT multiple-domain PRO included the Patient-Reported Outcome Measurement Information System (*PROMIS)-10*, yielding scores for global physical and global mental health [34]. Items were selected based on calibrations from large item banks for core domains of general health [34]. The CTT-based PRO was the 7-item *Environmental Mastery* subscale of the Ryff Psychological Well-Being measure [35]. The NeuroQOL and PROMIS-10 scores were computed using the raw score to T-score conversion tables, resulting in a standardized T-score metric (mean = 50, SD = 10) [33, 34]. The Ryff items are re-coded if negatively worded and summed to create a sum score [36]. Higher scores reflect better QOL on all measures.

Cognitive appraisal processes underlying responses to the patient-reported outcomes were assessed using the *QOL Appraisal Profile* – v2 [19]. This 85-item measure yields 12 orthogonal second-order component scores: Wellness Focus, Health Worries, Recent Challenges, Spiritual Focus, Relationship Focus, Maintain Roles, Independence, Reduce Responsibilities, Pursue Dreams, Anticipating Decline, Worry Free, and Lightness of Being. (See Additional file 1: Table S1 for details.) The interpretation of composite scores is a greater emphasis on the appraisal processes included in the component. In the case of negative versus positive loadings within a component, the interpretation would be one either attended to this pole (positive items) or tended to the opposite pole (negative items).

Demographic characteristics included year of birth, gender, ethnicity, race, cohabitation/marital status, with whom the person lives, employment status, annual household income categories, difficulty paying bills [37], and number of comorbidities, as measured by the *Self-Administered Comorbidity Questionnaire* [38]. Occupational complexity was assessed using questions querying the job that was closest to the respondent’s current or past occupation, which were then scored for complexity using the O*NET system. This comprehensive job-classification system from the National Center for O*NET Development provides in-depth classification of job complexity, with higher scores reflecting more training and skills required to perform that occupation [39].

We created catalyst variables reflecting significant life events since the baseline data collection. Positive and negative changes in four domains were created: marital, work, job-status, and comorbidity-burden. Positive marital change comprised going from being single to cohabiting or married, whereas negative marital change comprised going from single or married to separated, divorced or widowed. Positive work change comprised going from employed to retired; unemployed to employed or disabled; retired or disabled to employed. In contrast, negative work change comprised going from employed to unemployed or disabled. Positive job-status change comprised getting a promotion (i.e., increase in job complexity) over follow-up, whereas negative job-status change comprised getting demoted (i.e., decrease in job complexity). Comorbidity-burden change comprised reporting new comorbidities over follow-up. The positive- and negative-life event groups were kept distinct by domain in subsequent analyses.

### Statistical analysis

This analysis utilized the Rapkin and Schwartz’ regression residual modeling approach [18, 40] to investigate response-shift effects. This application of standard regression modeling refers to the specific sets of variables included in the first “standard” model, and the response-shift interpretation when the residual can be explained by change in appraisal. Building on a precedent for using residual modeling to study epiphenomena [41], we computed “standard model” regressions separately for the five PRO change scores (Global Physical Health, Global Mental Health, Applied Cognition, Well-Being, Environmental Mastery). These models adjust for the sociodemographic/medical characteristics generally considered relevant to QOL (antecedents in Fig. 1). This “standard” model adjusted for age, age at diagnosis, gender, education, ethnicity, income, received help to complete survey, employment status, marital status, and baseline number of comorbidities. The model residuals were saved, and subsequent models used these residualized change scores as dependent variables. This residual-modeling approach highlights the effect of change, controlling for baseline values.

To assess selection biases, t-tests or chi-squared analyses compared demographic characteristics in the attrition and analytic samples. To reduce the number of variables included in the subsequent multivariable analyses, analysis of variance (ANOVA) was used to compare each appraisal score as well as the raw and residualized PRO change scores for the catalyst groups. Contrast variables were created for Marital, Work, and O*NET change such that a “-1” reflected negative change; “0” reflected no change; and “+ 1” reflected a positive change. These dummy variables are coded to create contrasts for each concept measured so that the valence of the change is included in the dummy variable being analyzed. For comorbidity-burden change, a “1” reflected an increase and a score of “0” reflected no change.

The independent variables of the residualized-change analyses were catalyst groups, relevant appraisal domain identified by earlier ANOVAs, and their interaction if main effects were significant.. This paper is an exploratory analysis of a novel hypothesis. Accordingly, we have relaxed the Type I error rate to be 0.10 for deciding which variables to test in final models. Further, we have considered the results in light of what one would expect to find by chance (i.e., with a Type I error rate, one would expect to find 10% of comparisons to be “significant” by chance). Finally, we provide effect-size statistics for the above comparisons with conditional formatting and /or tabular footnotes to indicate effect-size magnitude as per Cohen ([42, 43]. Data analyses were implemented using Stata 15 [44].

## Results

### Sample

The study’s analytic sample included 1212 patients, 227 caregivers, and 42 patient-caregivers. Mean follow-up was 16.7 months (SD = 1.7; range: 13.5–25.7). Table 1 provides the sociodemographic characteristics and reported comorbidities of this heterogeneous sample.

**Table 1:** Sample Characteristics and Selection Bias Results

Baseline Variable	Attrition Sample^a^ (*n* = 2691)	Analytic Sample (*n* = 1481)	t-test *p*-value
Continuous Variables: Mean, SD	Mean SD	Mean SD	
Age	48.53 13.32	49.88 13.24	−3.15 **0.00**
Range	20~93	20~91	
Total Number of Comorbidities	3.89 2.33	3.94 2.28	−0.62 0.53
Range	3.80~3.98	3.82~4.06	
Categorical Variables: N, %	N %	N %	chi2 *p*-value
Respondent Type			
Patients	2111 78.5%	1212 81.8%	14.57 **0.00**
Caregivers	528 19.6%	227 15.3%	
Both patient and caregiver	52 19.3%	42 28.4%	
Gender			
Male	362 13.45	209 14.11	0.35 0.84
Female	2320 86.21	1269 85.69	
Other	5 0.19	3 0.2	
Co-morbidities			
Arthritis	1057 39.28	632 42.67	4.52 **0.03**
Asthma	498 18.51	294 19.85	1.12 0.29
Back Pain	1538 57.15	855 57.73	0.10 0.76
Cancer	1335 49.61	730 49.29	0.06 0.80
Depression	1365 50.72	712 48.08	2.74 0.10
Diabetes	317 11.78	168 11.34	0.17 0.68
Heart Disease	218 8.10	110 7.43	0.57 0.45
High Blood Pressure	719 26.72	429 28.97	2.57 0.11
Insomnia	1210 44.96	652 44.02	0.45 0.50
Kidney Disease	143 5.31	80 5.40	0.02 0.90
Liver Disease	115 4.27	56 3.78	0.59 0.44
Lung Disease	311 11.56	166 11.21	0.13 0.71
Stroke	91 3.38	42 2.84	0.90 0.34
Ulcer or Stomach Disease	439 16.31	207 13.98	3.88 **0.05**
Other	1110 41.25	688 46.46	9.26 **0.00**
Marital Status			
Single (never married)	368 13.68	211 14.25	1.61 0.90
Married	1627 60.46	899 60.7	
Cohabitation /Domestic Partner	159 5.91	100 6.75	
Divorced	60 2.23	33 2.23	
Separated	335 12.45	180 12.15	
Widowed	104 3.86	52 3.51	
Missing	38 1.41	6 0.41	
Ethnicity			
Not Hispanic or Latino	2439 90.64	1379 93.11	4.87 0.03
Hispanic or Latino	156 5.8	63 4.25	
Missing	96 3.57	39 2.63	
Race			
American Indian or Alaskan Native	80 2.97	35 2.36	1.32 0.25
Middle Eastern	8 0.30	6 0.41	0.33 0.56
South Asian	7 0.26	4 0.27	0.00 0.95
Other Asian	30 1.11	13 0.88	0.53 0.47
Black or African-America	142 5.28	59 3.98	3.48 *0.06*
Native Hawaiian, Other Pacific Islander	16 0.59	7 0.47	0.26 0.61
White	2413 89.67	1372 92.64	10.02 **0.00**
Education			
Less than High School	58 2.16	16 1.08	43.92 **0.00**
Graduated From High School or GED	714 26.53	299 20.19	
Some College or Technical School	519 19.29	259 17.49	
Graduated from College	803 29.84	484 32.68	
Postgraduate School or Degree	536 19.92	390 26.33	
Missing	61 2.27	33 2.23	
Employment Status at Baseline			
Working	1271 47.23	705 47.6	9.80 **0.02**
Currently Not working	365 13.56	156 10.53	
Retired (not due to ill health)	323 12	199 13.44	
Disabled and/or retired due to health	676 25.12	399 26.94	
Missing	56 2.08	22 1.49	
Occupational Complexity (O*NET Job Zone)			
1: Little or No Preparation Needed	115 4.27	60 4.05	21.53 **0.00**
2: Some Preparation Needed	529 19.66	252 17.02	
3: Medium Preparation Needed	877 32.59	439 29.64	
4: Considerable Preparation Needed	704 26.16	440 29.71	
5: Extensive Preparation Needed	271 10.07	203 13.71	
Missing	195 7.25	87 5.87	
Income			
Less than $15,000	247 9.18	123 8.31	5.39 0.50
$15,001 to $30,000	387 14.38	201 13.57	
$30,001 to $50,000	461 17.13	250 16.88	
$50,001 to $100,000	727 27.02	432 29.17	
$100,001 to $150,000	319 11.85	189 12.76	
$150,001 to $200,000	101 3.75	70 4.73	
Over $200,000	88 3.27	46 3.11	
Missing	361 13.42	170 11.48	
Difficulty Paying Bills^b^			
Not at all difficult	NA	528 35.65	
Slightly difficult	NA	334 22.55	
Somewhat difficult	NA	264 17.83	
Very difficult	NA	148 9.99	
Extremely difficult	NA	142 9.59	
Missing	NA	65 4.39	

^a^Attrition sample is missing follow-up data

^b^Information available at follow-up only

Bolded numbers reflect statistical signficance (i.e., *p*<0.05)

Selection-bias analyses revealed that the participants retained in the study were slightly older, less likely to be caregivers, more likely to have arthritis, and less likely to have an ulcer or stomach disease (Table 1). They were more likely to be non-Hispanic, White, and more educated; and to be/have been engaged in occupation requiring extensive preparation.

Table 2 shows the catalyst groups created for positive and negative significant life events. More people reported positive changes than negative changes on marital status and work change (ratio of 1.5 and 2.3, respectively). In contrast, job-status changes were equally divided among promotion and demotion. Overall, the life-changes reflected 3–35% of the sample, with the most prevalent being job-status change and the least prevalent being comorbidity-burden change.

**Table 2:** Catalyst Variables Created by Significant Life Event Change over Follow-up

Catalyst Domain	n	Positive: Negative	Proportion of Total Sample
Marital Status			
Positive Change (single to cohabiting or married)	74	1.51	0.08
Negative Change (single or married to separated, divorces or widowed)	49		
Work Change			
Positive Change (employed to retired; unemployed to employed disabled; retired or disabled to employed)	118	2.27	0.11
Negative Change (employed to unemployed or disabled)	52		
Job Status Change			
Positive Change (promotion, i.e., increase in job complexity)	256	0.96	0.35
Negative Change (demotion, i.e., decrease in job complexity)	266		
Comorbidity Burden Change			
Negative Change (Reported new comorbidities)	44	NA	0.03

The largest PRO changes were found in the PROMIS Global Mental Health, PROMIS Global Physical Health, and NeuroQOL Applied Cognition General Concerns (t = − 12.95, − 1.92, and 1.69, respectively; *p* < 0.0001, 0.03, and 0.05, respectively; Table 3), all suggesting deterioration on the PRO over time. The other PROs showed non-significant change over time. The appraisal change scores were also generally small, with the largest mean changes found in Health Worries and Recent Challenges. Of note, the standard deviation of the mean change scores were relatively large, as were the ranges, suggesting substantial change distributions across the variables of interest in the study sample.

**Table 3:** Change Scores on Patient-Reported Outcomes (post-minus-pre)

Measure	Change Score	Obs	Mean	Std. Dev.	Min	Max
QOL Appraisal Profile v2	Wellness Focus	1481	−0.23	2.49	−9.95	8.31
	Health Worries	1481	−0.97	4.90	−23.54	16.08
	Recent Challenges	1481	−0.59	4.40	−18.53	15.73
	Spiritual Focus	1481	0.13	1.72	−7.24	7.31
	Relationship Focus	1481	−0.14	2.01	−10.63	9.11
	Maintain Roles	1481	0.02	1.19	−4.27	4.11
	Independence	1481	0.07	1.42	−6.45	5.91
	Reduce Responsibilities	1481	0.01	1.08	−4.28	3.80
	Pursue Dreams	1481	0.09	1.47	−6.09	4.95
	Anticipating Decline	1481	−0.09	1.71	−8.47	6.76
	Worry-Free	1481	−0.09	1.36	−5.37	5.95
	Lightness of Being	1481	−0.08	1.28	−4.96	4.65
PROMIS 10	Global Physical Health Change Score	1481	−3.81	11.31	−57.70	32.80
	Global Mental Health Change Score	1481	−0.40	8.03	−62.50	40.50
NeuroQOL	Applied Cognition Change Score-General concerns	1464	−0.27	6.14	−32.40	29.00
	Applied Cognition Change Score-Executive function	1464	−0.08	5.39	−36.80	24.80
	Positive Affect and Well-Being Change Score	1462	0.11	5.94	−41.70	31.50
Ryff Psychological Well-Being	Environmental Mastery	1462	−0.10	12.26	−50.37	54.63

Different appraisal-change scores were relevant to the catalyst groups (Table 4). People who married or started an intimate relationship placed a greater emphasis on their legacy and generativity, as well as their degree of independence. People who either gained employment or lost employment placed a greater emphasis on relationships, and people who gained employment placed substantially less emphasis on maintaining roles. People who had a larger comorbidity burden at follow-up tended to indicate that their ability to reduce responsibilities was a much less important consideration in rating their QOL. None of the raw or residualized change scores on the IRT single-domain or –general PROs was associated with any of the catalyst groups, but the Ryff Environmental Mastery raw change score was associated with work change (*p* < 0.02).

**Table 4:** ANOVA Results Comparing Catalyst Groups across PRO Change Scores

Change Score (post-pre)	Any Marital Change			Any Work Change			Any Job-Status Change			Increased Comorbidity change		
	F	*p*-value	Eta squared	F	*p*-value	Eta squared	F	*p*-value	Eta squared	F	*p*-value	Eta squared
Appraisal Change												
Wellness Focus	2.13	0.12	0.003	0.07	0.93	0.000	1.57	0.21	0.003	0.78	0.38	0.001
Health Worries	0.71	0.49	0.001	1.75	0.17	0.002	0.24	0.78	0.000	0.04	0.84	0.000
Recent Challenges	0.21	0.81	0.000	1.15	0.32	0.002	0.07	0.94	0.000	0.01	0.90	0.000
Spiritual Focus	3.75	**0.02**	0.005	1.91	0.15	0.003	0.09	0.92	0.000	0.67	0.41	0.000
Relationship Focus	0.23	0.80	0.000	4.63	**0.01**	0.006	0.50	0.61	0.001	0.43	0.51	0.000
Maintain Roles	1.66	0.19	0.002	3.62	**0.03**	0.005	0.31	0.73	0.001	0.82	0.37	0.001
Independence	3.07	**0.05**	0.004	1.34	0.26	0.002	1.01	0.36	0.002	1.76	0.19	0.001
Reduce Responsibilities	0.19	0.83	0.000	2.28	0.10	0.003	0.38	0.68	0.001	4.31	**0.04**	0.003
Pursue Dreams	1.32	0.27	0.002	0.68	0.51	0.001	0.90	0.41	0.002	1.70	0.19	0.001
Anticipating Decline	0.30	0.74	0.000	0.39	0.68	0.001	0.70	0.50	0.001	0.11	0.74	0.000
Worry-Free	0.16	0.85	0.000	0.21	0.81	0.000	0.24	0.79	0.000	0.72	0.40	0.000
Lightness of Being	1.84	0.16	0.003	0.36	0.70	0.000	0.21	0.81	0.000	1.36	0.24	0.001
Raw Change Score												
PROMIS Global Physical Health	0.21	0.81	0.000	0.22	0.80	0.000	1.87	0.15	0.003	1.15	0.28	0.001
PROMIS Global Mental Health	0.04	0.96	0.000	1.98	0.14	0.003	1.69	0.18	0.003	0.00	1.00	0.000
NeuroQOL Applied Cognition-General Concerns	0.54	0.58	0.001	1.24	0.29	0.002	0.65	0.52	0.001	0.04	0.85	0.000
NeuroQOL Applied Cognition-Executive Function	0.56	0.57	0.001	0.11	0.90	0.000	0.02	0.98	0.000	1.10	0.29	0.001
NeuroQOL Positive Affect & Well-Being	0.01	0.99	0.000	1.48	0.23	0.002	0.88	0.41	0.002	0.64	0.42	0.000
Ryff Environmental Mastery	1.21	0.30	0.002	3.71	**0.02**	0.005	1.45	0.23	0.003	1.95	0.16	0.001
Residualized Change Score												
PROMIS Global Physical Health	0.03	0.97	0.000	0.06	0.94	0.000	1.26	0.28	0.003	0.38	0.54	0.000
PROMIS Global Mental Health	0.51	0.60	0.001	2.15	0.12	0.004	2.14	0.12	0.005	0.00	0.95	0.000
NeuroQOL Applied Cognition-General Concerns	1.02	0.36	0.002	1.58	0.21	0.003	0.26	0.77	0.001	0.18	0.67	0.000
NeuroQOL Applied Cognition-Executive Function	0.39	0.68	0.001	0.01	0.99	0.000	0.33	0.72	0.001	1.14	0.29	0.001
NeuroQOL Positive Affect & Well-Being	0.10	0.91	0.000	0.75	0.47	0.001	0.54	0.58	0.001	0.95	0.33	0.001
Ryff Environmental Mastery	1.71	0.18	0.003	2.13	0.12	0.004	1.29	0.28	0.003	1.68	0.19	0.001

Note: Eta-Squared effect sizes are all less than Small as defined by Cohen (1988)

Bolded numbers reflect statistical signficance (i.e., *p*<0.05)

Additional file 2: Table S2 shows the results of ANOVA models testing the sensitivity to catalysts of individual items’ change scores for each PRO. Although only the Ryff score was associated with a catalyst, item-level analyses revealed more sensitivity to catalysts among the PROs. The PROMIS-10 and the Ryff Environmental Mastery subscale had the largest proportion of items associated with catalysts at 23% and 18%, respectively (using a Type 1 error rate of 0.10, the probability of exactly this proportion of significant associations is 0.01 and 0.09). In contrast, the NeuroQOL Positive Affect & Well-Being and Applied Cognition items had relatively few items associated with the catalysts at 8% and 6%, respectively. Thus the CTT-based and IRT multiple-domain measures had more associations than expected, whereas the IRT single-domain measures had less-than-expected. These findings support the hypothesis that CTT-based and IRT multiple-domain measures are most sensitive to response-shift effects, in contrast with IRT single-domain measures.

Additional file 2: Table S3 shows the alpha coefficients for the baseline, follow-up, and change scores for PROs. The IRT single-domain measures had the highest alpha coefficients for baseline, follow-up, and change scores. The IRT multiple domain and CTT-based measures had slightly lower but still high alpha coefficients at baseline and follow-up, but substantially lower alpha for change scores.

### Residual modeling of response shift

In models predicting residualized change in global physical health, none of the catalysts was associated with longitudinal trajectories (Table 5). Appraisal changes were, however, significant predictors of residualized change in Global Physical Health. Specifically, increased endorsement of Independence appraisal was associated with improved Global Physical Health, after adjusting for positive and negative marital changes. Increased concern about Maintaining Roles was associated with worse Global Physical Health, after adjusting for positive and negative work change. Increased endorsement of Reduce Responsibilities was associated with worse Global Physical Health, after adjusting for increases in comorbidities. Job-status change was unrelated.

**Table 5:**
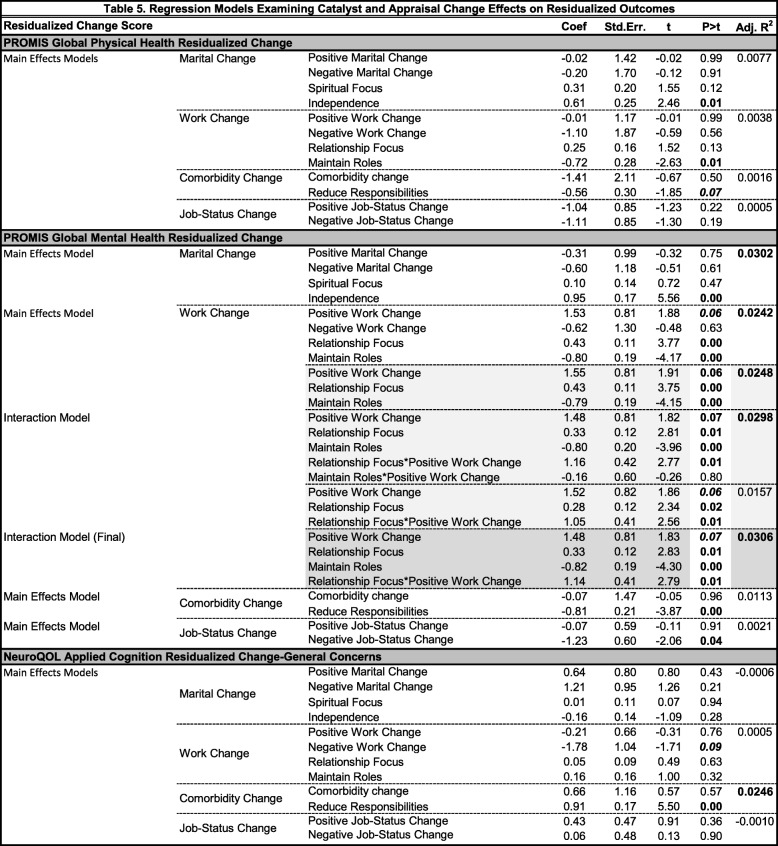

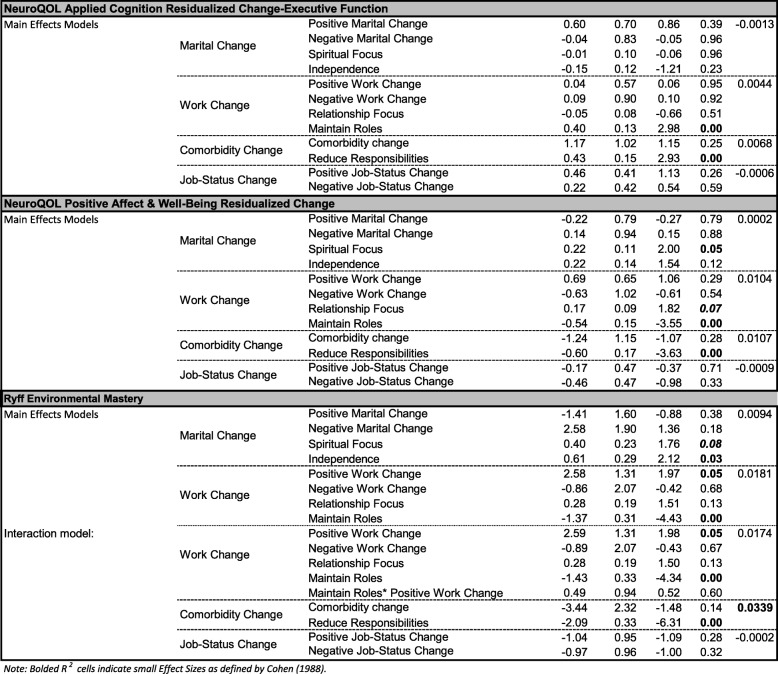
Regression Models Examining Catalyst and Appraisal Change Effects on Residualized Outcomes

In models predicting residualized change in Global Mental Health, positive work change and negative job-status change were associated with longitudinal trajectories (Table 5). Positive work change had a trend association with improved Global Mental Health over time. Further, an increased focus on Relationships and a decreased focus on Maintain[ing] Roles were associated with improved global mental health over time. There was a significant interaction effect between the Positive Work Change catalyst and change in Relationship Focused appraisal, suggesting that people who had both positive work changes and an increased focus on relationships reported better Global Mental Health over time (Additional file 1: Figure S1). Finally, negative job-status change was associated with worsened Global Mental Health, and appraisal change had no impact.

In the models predicting residualized Global Mental Health after adjusting for marital change and comorbidity change, appraisal change was associated with residualized global mental health (Table 5). An increased endorsement of Independence appraisals was associated with better Global Mental Health, after adjusting for positive and negative marital changes; and a decreased endorsement of Reduce Responsibilities appraisals was associated with better Global Mental Health, after adjusting for increases in comorbidities.

In models predicting residualized change in cognitive functioning, no catalysts were associated but appraisal changes were (Table 5). An increased emphasis on Relationships, Maintain[ing] Roles (trend), and Reduc[ing] Responsibilities were associated with improved reported cognitive function over time, after adjusting for the relevant catalyst groups (work and comorbidity change, respectively). Job-status change was unrelated.

In models predicting residualized change in positive affect and well-being, no catalysts were associated but appraisal changes were (Table 5). An increased emphasis on Spiritual Focus and Relationships (trend), and a decreased emphasis on Maintain[ing] Roles and Reduc[ing] Responsibilities were all associated with improved well-being over time, after adjusting for the relevant catalyst groups (marital, work, and comorbidity change, respectively). Job-status change was unrelated.

In models predicting residualized change in Environmental Mastery, an increased Spiritual Focus and Independence, and a decreased focus on Maintain[ing] Roles and Reduc[ing] Responsibilities were associated with improved Environmental Mastery. Positive work change and change in Maintaining-Roles appraisal were associated with improved Environmental Mastery (Table 5), supporting a direct response-shift effect.

**Fig. 3:**
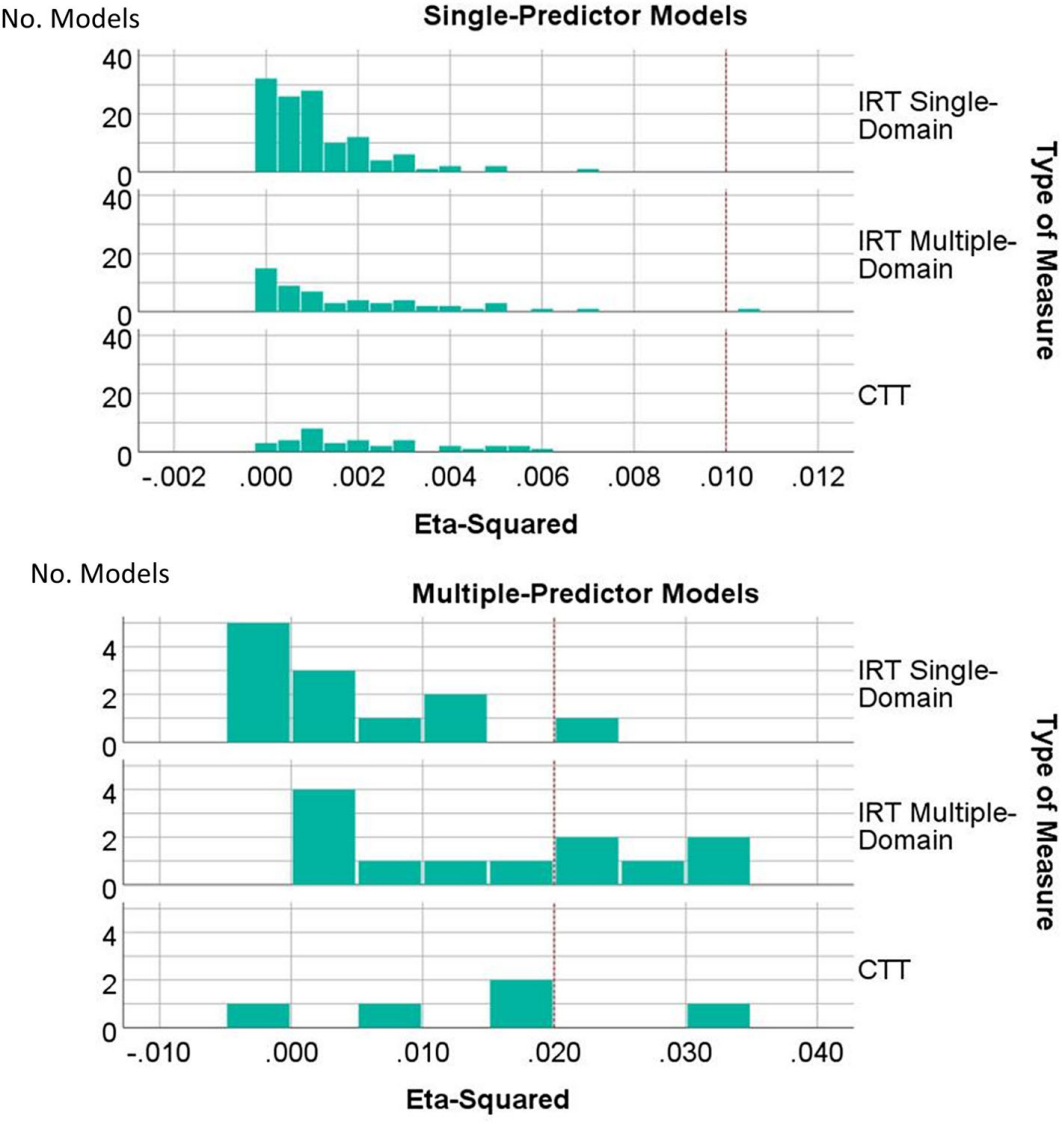
Distribution of effect sizes by type of measure for single-predictor models (top panel) and multiple-predictor models (bottom panel). Effects were generally small, and were systematically smaller for the IRT Single-Domain Measures. The IRT Multiple-Domain and CTT-Based measures were more likely to achieve effect size that met or exceeded Cohen’s [43] criteria for small effects. Dashed vertical line indicates cut-point of eta-squared ≥0.01 for single-predictor models, and ≥ 0.02 for multiple predictor models

Figure 3 summarizes the effect sizes across the univariable and multivariable models, contrasting the three types of measures. Although effects were generally small, they were systematically smaller for the IRT Single-Domain Measures (ANOVA F = 8.35, df = 2, 242, *p* < 0.001, eta-squared = 0.065). The IRT Multiple-Domain and CTT-Based measures were more likely to achieve effect sizes that met or exceed Cohen’s [43] criteria for small effects.

## Discussion

Our findings suggest that PROs developed in different ways and/or with different goals may be differentially sensitive to response-shift effects. The IRT single-domain measures evaluated in this study were not impacted by major life events at the item- or scale-level. In contrast, the IRT multiple domain and CTT-based measures were more sensitive to such catalysts. For the IRT multiple-domain measure, response shift was evidenced only at the item level, whereas the CTT-based measure demonstrated this sensitivity at both the item- and scale-level.

Our findings suggest that response-shift effects are present and detectable using a direct measure of changes in appraisal. The catalyst variables were associated with different appraisal process trajectories. These associations had face validity, i.e., they made sense. Marital change was associated with an increased focus on legacy or generativity and independence, whereas work change was associated with an increased focus on relationships and a decreased focus on role maintenance. Increased comorbidity burden was associated with a decreased emphasis on reducing responsibilities.

The IRT single-domain measures evaluated in this study seemed to change in the same way over time (i.e., in lock-step). In contrast, the IRT-general and CTT measures’ items changed in much less of a lock-step fashion. Thus, compared to IRT single-domain measures, IRT multiple domain and CTT-based measures could be more sensitive to response-shift effects over time. They have lower stability over time because different appraisal processes apparently influence subscale items differentially.

The implications of the study findings are that appraisal processes – and response shift effects – are relevant and influence the interpretation of change even for IRT single-domain measures. The IRT multiple domain global health measure and the CTT-based Ryff subscale were sensitive to life events *and* to appraisal processes.

Despite the present study’s notable strengths (i.e., large heterogeneous sample, longitudinal data), its limitations must be acknowledged. First, several factors prevent us from making definitive statements about how IRT/CTT measures are differentially responsive to catalyst and response-shift effects. The findings were generally small effect sizes, some of which may reflect weak operationalization of catalysts. We cannot know the true valence of the catalysts from the perspective of the respondent (e.g., divorce can be a positive change for some, retirement can be a challenging transition for others). Small samples of exemplar measures also limit our ability to generalize. The results could be due to peculiarities of the scales used, not necessarily the methods used to create them. Further, although IRT-developed measures might generally emphasize unidimensionality and are stricter and more rigorous than CTT-developed measures, this generalization that may not always apply. For example, the PROMIS and NeuroQOL item banks reportedly balanced items that were “unidimensional enough” with items deemed clinically important. Finally, other sources of measurement error could be at play. For example, the small sample size of change groups limited our statistical power to detect response-shift effects. Therefore, the evidence should be considered preliminary, and future research should attempt to replicate the study with a larger sample of IRT- and CTT-based measures, explicit measurement of the valence of catalysts, and larger sample sizes within catalyst and no-catalyst groups.

In summary, our findings highlight several under-appreciated notions about QOL measurement. Even when item difficulty and scale unidimensionality are constant, the construct to which they refer may change subjective meaning. Differences in appraisal are related to all of the types of measures regardless of their provenance, and are not a form of bias. It might be tempting to consider creating scales that are not subject to response shift due to changes in appraisal. This would, however, neither be feasible nor useful. The only way to reduce differences in the implicit meaning and context that individuals read into items would be to add detailed instructions to constrain their ways of thinking. This would not only be cumbersome; it would distort our understanding of individuals’ actual experience. Direct assessment of changes in the cognitive criteria that people use to evaluate their QOL is far more fruitful. How individuals appraise QOL is as interesting and important as the numerical rating of QOL that they provide.

## Conclusions

Our findings suggest that response-shift effects are present and detectable using a direct measure of changes in appraisal. PROs developed in different ways and/or with different goals may be differentially sensitive to response-shift effects. The IRT single-domain measures evaluated in this study were not impacted by major life events at the item- or scale-level. In contrast, the IRT multiple domain and CTT-based measures were more sensitive to such catalysts. For the IRT multiple-domain measure, response shift was evidenced only at the item level, whereas the CTT-based measure demonstrated this sensitivity at both the item- and scale-level. The implications of the study findings are that appraisal processes – and response shift effects – are relevant and influence the interpretation of change even for IRT single-domain measures. This study is the first to address this research question, so its findings are preliminary and suggestive and should be replicated in studies with more measures of each type.

## Supplementary information

**Additional file 1: Figure S1.** Moderated response-shift effect. This figure illustrates the significant interaction effect between Positive Work Change and Relationship Focus in predicting residualized change in global mental health. Thus, people who had both positive work changes and an increased relationship focus tended to have notably better global mental health over time, after adjusting for changes in their focus on maintaining roles.

**Additional file 2: Table S1.** Description of QOLAPv2 Component Scores. **Table S2.** ANOVA Results Comparing Catalyst Groups for PRO Change Items. **Table S3.** Alpha Reliability Coefficients for PROs at Baseline, Follow-up and Change scores.

## Data Availability

The study data are confidential and thus not able to be shared.

## Abbreviations

ANOVA: Analysis of variance
CTT: Classical test theory
DIF: Differential item functioning
HIPAA: Health Insurance Portability and Accountability Act
IRT: Item Response Theory
PRO: Patient-reported outcome
PROMIS: Patient-Reported Outcome Measurement Information System
QOL: Quality-of-life
