# Supplementary figures and images for: Does response shift impact interpretation of change even among scales developed using item response theory?
Source: J Patient Rep Outcomes. 2020 Jan 23;4:8. doi: 10.1186/s41687-019-0162-x (PMC6977794; doi:10.1186/s41687-019-0162-x)

## Slide 1
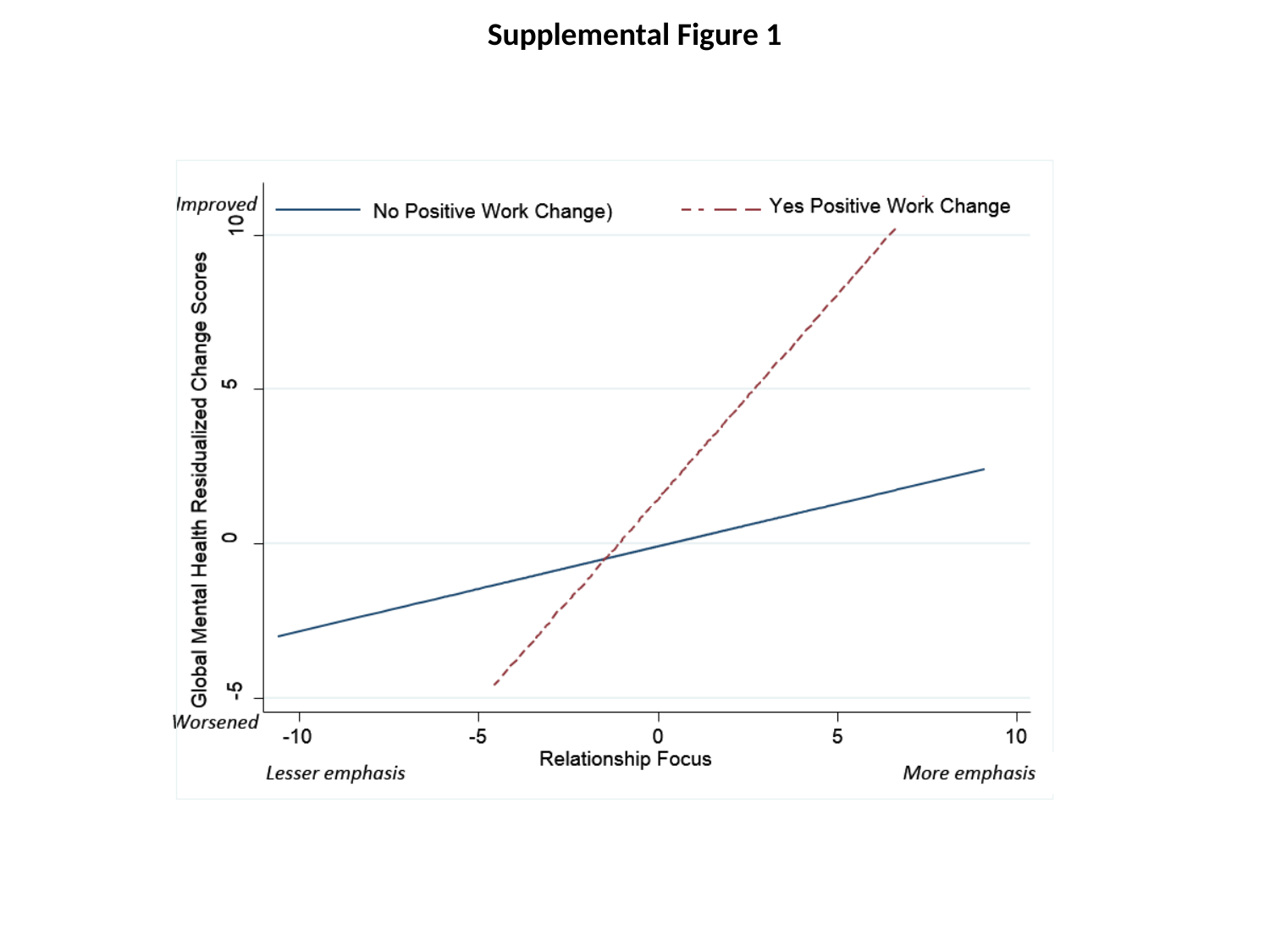

Supplemental Figure 1
#

Supplement: Supplementary file 1 — Additional file 1: Figure S1. Moderated response-shift effect. This figure illustrates the significant interaction effect between Positive Work Change and Relationship Focus in predicting residualized change in global mental health. Thus, people who had both positive work changes and an increased relationship focus tended to have notably better global mental health over time, after adjusting for changes in their focus on maintaining roles. [file 41687_2019_162_MOESM1_ESM.pptx]
