# Supplementary material for: Does response shift impact interpretation of change even among scales developed using item response theory?
Source: J Patient Rep Outcomes. 2020 Jan 23;4:8. doi: 10.1186/s41687-019-0162-x (PMC6977794; doi:10.1186/s41687-019-0162-x)
Supplement: Supplementary file 2 — Additional file 2: Table S1. Description of QOLAPv2 Component Scores. Table S2. ANOVA Results Comparing Catalyst Groups for PRO Change Items. Table S3. Alpha Reliability Coefficients for PROs at Baseline, Follow-up and Change scores. [file 41687_2019_162_MOESM2_ESM.pdf]

Table S1. Description of QOLAPv2 Component Scores

Second-Order Component Name	Meaning of QOL	Goals	Experience Sampling	Standards of Comparison	Combinatory Algorithm	First-Order Components Included Description	Total Variance Explained
1 Wellness Focus		x	x	x		Calm, healthy lifestyle, self acceptance, keep up activities and health care, focused on improvements, used to how things are, remain positive and balanced - do not think of the worst moments	6.6
2 Health Worries		x	x	x		Health worries - concern about what doctors say, high frequency of social comparison	6.1
3 Recent Challenges		x	x		x	Recall relevant episodes and recent challenges, accept people, let go of self-expectations, make multiple comparisons	5.9
4 Spiritual Focus	x	x				Faith and generativity	5.1
5 Relationship Focus	x	x				Romance improved relationships, self-acceptance	4.7
6 Maintain Roles	x	x				Accomplishments and maintaining community and work roles (versus getting rid of family problems, self-acceptance, calm, no regrets)	4.6
7 Independence	x	x				Independence - resolve problems - stay at home - no regrets, resolve recent money problems and other negative circumstances, keep active and fully participate	4.5
8 Reduce Responsibilities	x	x	x			Let go of responsibilities for house, others, self-expectations, spend time with family, influence by questionnaire	4.2
9 Pursue Dreams		x		x		Pursue dreams and goals, change living situation versus focus on comparisons to others my age and stay in current living situation,	4.2
10 Anticipating Decline		x			x	Prepare loved ones and living situations for declines - ups and downs, compare self to what MD told them	4.0
11 Worry-Free		x		x		Compare to others without health limits versus those who have had similar illness, be worry free, solve money, living, practical problems versus accept people and roles, let go of self-expectations	3.9
12 Lightness of Being			x	x		Spontaneous - not complain - how I saw myself before illness, how others see me	3.8

Table S2. ANOVA Results Comparing Catalyst Groups for PRO Change Items

Change Items (post-pre)	Marital Change			Work Change			Job-Status Change			Comorbidity change			Proportion associated with catalysts†
	F	p	Eta-Squared	F	p	Eta-Squared	F	p	Eta-Squared	t	p	Eta-Squared	
**PROMIS-10 items**													
**Physical Functioning Items**													
In general, how would you rate your physical health?	0.43	0.65	0.0004	0.80	0.45	0.0011	0.64	0.53	0.0012	0.73	0.47	0.0004	
To what extent are you able to carry out your everyday physical activities such as walking, climbing stairs, carrying groceries, or moving a chair?	0.36	0.70	0.0006	4.93	**0.01**	0.0069	0.96	0.38	0.0018	-0.95	0.34	0.0006	
In the past 7 days, how would you rate your fatigue on average?	0.32	0.73	0.0005	0.18	0.83	0.0003	0.07	0.93	0.0001	0.88	0.38	0.0005	
In the past 7 days, how would you rate your pain on average?	1.59	0.20	0.0038	3.61	**0.03**	0.0050	5.95	**0.00**	0.0107	-2.36	**0.02**	0.0038	
**Social Functioning items**													
In general, please rate how well you carry out your usual social activities and roles. (This includes activities at home, at work and in your community, and responsibilities as a parent, child, spouse, employee, friend, etc.).	1.32	0.27	0.0023	3.61	**0.03**	0.0051	1.26	0.28	0.0023	-1.84	**0.07**	0.0023	0.23
**Emotional Functioning Items**													
In general, would you say your quality of life is:	0.11	0.89	0.0004	1.49	0.23	0.0021	3.37	**0.03**	0.0061	-0.75	0.45	0.0004	
In general, how would you rate your mental health, including your mood and your ability to think?	0.15	0.86	0.0001	1.08	0.34	0.0015	1.41	0.25	0.0026	0.36	0.72	0.0001	
In general, how would you rate your satisfaction with your social activities and relationships?	1.22	0.30	0.0001	2.52	**0.08**	0.0035	0.09	0.92	0.0002	0.30	0.76	0.0001	
In the past 7 days, how often have you been bothered by emotional problems such as feeling anxious, depressed or irritable?	0.40	0.67	0.0008	1.12	0.33	0.0016	0.56	0.57	0.0010	1.10	0.27	0.0008	
In general, would you say your health is:	3.28	**0.04**	0.0001	0.83	0.44	0.0012	1.91	0.15	0.0035	-0.40	0.69	0.0001	
**NeuroQOL Applied Cognition Items**													
**General Concerns Items**													
I had to read something several times to understand it.	1.31	0.27	0.0018	0.18	0.84	0.0002	0.55	0.58	0.0010	-0.40	0.69	0.0001	
I had trouble keeping track of what I was doing if I was interrupted.	2.03	0.13	0.0028	0.30	0.74	0.0004	1.61	0.20	0.0029	1.05	0.29	0.0008	
I had difficulty doing more than one thing at a time.	0.33	0.72	0.0005	0.76	0.47	0.0011	1.42	0.24	0.0026	-0.43	0.67	0.0001	
I had trouble remembering new information, like phone numbers of simple instructions.	1.48	0.23	0.0020	0.11	0.90	0.0002	0.05	0.95	0.0001	-0.03	0.98	0.0000	
I had trouble thinking clearly.	1.12	0.33	0.0015	2.97	**0.05**	0.0041	0.19	0.83	0.0003	0.93	0.35	0.0006	
My thinking was slow.	0.26	0.77	0.0004	1.83	0.16	0.0025	0.28	0.76	0.0005	-0.22	0.82	0.0000	
I had to work really hard to pay attention or I would make a mistake.	0.89	0.41	0.0012	0.80	0.45	0.0011	1.41	0.24	0.0026	0.21	0.83	0.0000	
I had trouble concentrating.	1.33	0.26	0.0018	0.86	0.42	0.0012	0.97	0.38	0.0018	-1.43	0.15	0.0014	
**Executive Function Items**													
Checking the accuracy of financial documents, (e.g. bills, checkbook, or bank statements)?	0.34	0.71	0.0005	0.19	0.83	0.0003	0.20	0.81	0.0004	0.03	0.98	0.0000	0.06
Counting the correct amount of money when making purchases?	5.01	**0.01**	0.0070	0.33	0.72	0.0005	0.23	0.79	0.0004	0.10	0.92	0.0000	
Reading and following complex instructions (e.g. directions for a new medication)?	2.41	**0.09**	0.0033	0.49	0.61	0.0007	1.06	0.35	0.0019	-1.73	**0.08**	0.0021	
Planning for and keeping appointments that are not part of your weekly routine (e.g. a therapy or doctor's appointment, or a social gathering with friends and family)?	0.53	0.59	0.0007	1.12	0.33	0.0016	0.28	0.75	0.0005	-0.08	0.94	0.0000	
Managing your time to do most of your daily activities?	0.18	0.84	0.0002	0.09	0.91	0.0001	0.66	0.52	0.0012	-1.00	0.32	0.0007	

Change Items (post-pre)	Marital Change			Work Change			Job-Status Change			Comorbidity change			Proportion associated with catalysts†
	F	p	Eta-Squared	F	p	Eta-Squared	F	p	Eta-Squared	t	p	Eta-Squared	
Taking care of complicated tasks like managing a checking account or getting appliances fixed?	1.07	0.34	0.0015	0.20	0.82	0.0003	0.41	0.67	0.0007	-0.69	0.49	0.0003	
Keeping important personal papers such as bills, insurance documents, and tax forms organized?	1.86	0.16	0.0026	1.06	0.35	0.0015	0.54	0.59	0.0010	-0.78	0.44	0.0004	
Learning new tasks or instructions?	0.87	0.42	0.0012	0.36	0.69	0.0005	0.28	0.76	0.0005	-0.48	0.63	0.0002	
**NeuroQOL Positive Affect and Well-Being**													
I had a sense of well-being.	0.20	0.82	0.0003	0.09	0.92	0.0001	0.95	0.39	0.0017	0.60	0.55	0.0002	
I felt hopeful.	0.45	0.64	0.0006	1.16	0.31	0.0016	1.22	0.30	0.0022	0.41	0.68	0.0001	
My life was satisfying.	0.76	0.47	0.0011	1.14	0.32	0.0016	2.87	**0.06**	0.0052	1.25	0.21	0.0011	
My life had purpose.	0.87	0.42	0.0012	2.77	**0.06**	0.0039	0.47	0.63	0.0009	0.20	0.84	0.0000	
My life had meaning.	1.14	0.32	0.0016	3.40	**0.03**	0.0048	0.03	0.97	0.0000	-0.41	0.69	0.0001	0.08
I felt cheerful.	0.80	0.45	0.0011	2.11	0.12	0.0029	0.98	0.38	0.0018	0.94	0.35	0.0006	
My life was worth living.	0.79	0.46	0.0011	0.87	0.42	0.0012	0.44	0.65	0.0008	-0.07	0.94	0.0000	
I had a sense of balance in my life.	2.04	0.13	0.0028	0.38	0.69	0.0005	0.12	0.88	0.0002	0.72	0.47	0.0004	
Many areas of life were interesting to me.	1.14	0.32	0.0016	2.26	0.11	0.0032	0.04	0.96	0.0001	0.09	0.93	0.0000	
**Ryff Environmental Mastery**													
In general, I feel I am in charge of the situation in which I live.	0.80	0.45	0.0011	1.42	0.24	0.0020	1.59	0.20	0.0029	0.48	0.63	0.0002	
The demands of everyday life often get me down.	3.82	**0.02**	0.0053	1.46	0.23	0.0020	0.75	0.47	0.0014	1.64	0.10	0.0018	
I do not fit in well with the people and the community around me.	1.50	0.22	0.0021	4.12	**0.02**	0.0058	2.30	0.10	0.0042	-0.32	0.75	0.0001	
I am quite good at managing the many responsibilities of my daily life.	0.54	0.58	0.0008	1.78	0.17	0.0025	0.08	0.92	0.0001	0.83	0.41	0.0005	0.18
I often feel overwhelmed by my responsibilities.	3.07	**0.05**	0.0043	0.22	0.80	0.0003	2.71	**0.07**	0.0049	1.14	0.26	0.0009	
I have difficulty arranging my life in a way that is satisfying to me.	3.86	**0.02**	0.0054	0.60	0.55	0.0008	0.41	0.66	0.0008	0.61	0.54	0.0003	
I have been able to build a home and a lifestyle for myself that is much to my liking.	0.55	0.58	0.0008	2.19	0.11	0.0031	0.58	0.56	0.0011	0.71	0.48	0.0004	

† *Using a 0.10 cutoff, one would expect 10% would be significant by chance.Bolded values have p-values < 0.10.*

Table S3. Alpha Reliability Coefficients for PROs at Baseline, Follow-up and Change scores			
	Unstandardized Alpha Coefficient†		
	Baseline	Follow-Up	Change score
**NeuroQOL Cognition: General Concerns**			
I had to read something several times to understand it.	0.96	0.96	0.89
I had trouble keeping track of what I was doing if I was interrupted.	0.96	0.96	0.88
I had difficulty doing more than one thing at a time.	0.96	0.96	0.88
I had trouble remembering new information, like phone numbers of simple instructions.	0.96	0.96	0.88
I had trouble thinking clearly.	0.95	0.95	0.87
My thinking was slow.	0.95	0.95	0.87
I had to work really hard to pay attention or I would make a mistake.	0.95	0.95	0.88
I had trouble concentrating.	0.95	0.95	0.88
Test Scale	0.96	0.96	0.89
**NeuroQOL Cognition: Executive Function**			
Checking the accuracy of financial documents, (e.g. bills, checkbook, or bank statements)?	0.92	0.92	0.80
Counting the correct amount of money when making purchases?	0.92	0.93	0.81
Reading and following complex instructions (e.g. directions for a new medication)?	0.92	0.92	0.80
Planning for and keeping appointments that are not part of your weekly routine (e.g. a therapy or doctor's appointment, or a social gathering with friends and family)?	0.92	0.92	0.80
Managing your time to do most of your daily activities?	0.92	0.93	0.80
Taking care of complicated tasks like managing a checking account or getting appliances fixed?	0.91	0.92	0.79
Keeping important personal papers such as bills, insurance documents, and tax forms organized?	0.92	0.92	0.80
Learning new tasks or instructions?	0.92	0.92	0.79
Test Scale	0.96	0.96	0.89
**NeuroQOL Positive Affect and Well-Being**			
I had a sense of well-being.	0.95	0.95	0.87
I felt hopeful.	0.95	0.95	0.87
My life was satisfying.	0.94	0.95	0.86
My life had purpose.	0.94	0.95	0.86
My life had meaning.	0.94	0.95	0.86
I felt cheerful.	0.95	0.95	0.87
My life was worth living.	0.95	0.95	0.87

I had a sense of balance in my life.	0.95	0.95	0.87
Many areas of life were interesting to me.	0.95	0.95	0.87
Test Scale	0.96	0.96	0.89
**PROMIS-10 Global Physical Health**			
In general, how would you rate your physical health?	0.74	0.69	0.32
To what extent are you able to carry out your everyday physical activities such as walking, climbing stairs, carrying groceries, or moving a chair?	0.74	0.65	0.28
In the past 7 days, how would you rate your pain on average?	0.77	0.79	0.46
In the past 7 days, how would you rate your fatigue on average?	0.77	0.69	0.29
Test Scale	0.81	0.75	0.39
**PROMIS-10 Global Mental Health**			
In general, would you say your quality of life is:	0.78	0.81	0.55
In general, how would you rate your mental health, including your mood and your ability to think?	0.74	0.77	0.48
In general, how would you rate your satisfaction with your social activities and relationships?	0.78	0.80	0.55
In the past 7 days, how often have you been bothered by emotional problems such as feeling anxious, depressed or irritable?	0.81	0.83	0.57
Test Scale	0.82	0.84	0.61
**Ryff Environmental Mastery**			
In general, I feel I am in charge of the situation in which I live.	0.80	0.82	0.53
The demands of everyday life often get me down.	0.79	0.81	0.50
I do not fit in well with the people and the community around me.	0.81	0.83	0.54
I am quite good at managing the many responsibilities of my daily life.	0.80	0.83	0.55
I often feel overwhelmed by my responsibilities.	0.81	0.81	0.52
I have been able to build a home and a lifestyle for myself that is much to my liking.	0.79	0.82	0.54
I have difficulty arranging my life in a way that is satisfying to me.	0.79	0.80	0.51
Test Scale	0.82	0.84	0.57

† *Alpha coefficient for individual items reflects the deleted alpha coefficient, i.e., the scale-level alpha when this item is not included.*
